# The Role of Fibrosis and Liver-Associated Fibroblasts in the Pathogenesis of Hepatocellular Carcinoma

**DOI:** 10.3390/ijms20071723

**Published:** 2019-04-07

**Authors:** Jacopo Baglieri, David A. Brenner, Tatiana Kisseleva

**Affiliations:** 1 Department of Medicine, UC San Diego, La Jolla, CA 92093, USA; 2 Department of Surgery, UC San Diego, La Jolla, CA 92093, USA

**Keywords:** hepatocellular carcinoma (HCC), fibrosis, cancer-associated fibroblasts (CAFs), hepatic stellate cells (HSCs), tumor microenvironment

## Abstract

Hepatocellular carcinoma (HCC) is one of the most aggressive types of cancer and lacks effective therapeutic approaches. Most HCC develops in the setting of chronic liver injury, hepatic inflammation, and fibrosis. Hepatic stellate cells (HSCs) and cancer-associated fibroblasts (CAFs) are key players in liver fibrogenesis and hepatocarcinogenesis, respectively. CAFs, which probably derive from HSCs, activate into extracellular matrix (ECM)-producing myofibroblasts and crosstalk with cancer cells to affect tumor growth and invasion. In this review, we describe the different components which form the HCC premalignant microenvironment (PME) and the tumor microenvironment (TME), focusing on the liver fibrosis process and the biology of CAFs. We will describe the CAF-dependent mechanisms which have been suggested to promote hepatocarcinogenesis, such as the alteration of ECM, CAF-dependent production of cytokines and angiogenic factors, CAF-dependent reduction of immuno-surveillance, and CAF-dependent promotion of epithelial-mesenchymal transition (EMT). New knowledge of the fibrosis process and the role of CAFs in HCC may pave the way for new therapeutic strategies for liver cancer.

## 1. Introduction

Hepatocellular carcinoma (HCC) is one of the most aggressive and fastest growing malignancies [1]. HCC is only second to lung cancer as a leading cause of cancer-related death worldwide [2]. Since, in most patients, HCC is diagnosed at a late stage, therapeutic treatments are limited and the five-year survival rate is less than 12% [3]. Most HCC cases are in southeast Asia, where the major cause of HCC is chronic hepatitis B virus (HBV) infections. In contrast, in sub-Saharan Africa, the main risk factor is exposure to aflatoxin B [4]. However, in Japan, North America, and Europe, major causes of HCC are hepatitis C virus (HCV) infections, alcoholic liver disease, and non-alcoholic fatty liver disease (NAFLD) [4]. This chronic liver injury causes liver fibrosis, which is characterized by the activation of hepatic stellate cells (HSCs) into extracellular matrix (ECM)-producing myofibroblasts [5,6,7]. In chronic liver injury, continuous accumulation of ECM results in the progressive substitution of the liver parenchyma by scar tissue. Regardless of the etiology of liver injury, HCC is strongly associated with liver fibrosis and cirrhosis, with about 80–90% of HCC cases having underlying fibrosis [8], and approximately one in three patients with cirrhosis will develop HCC in their lifetime [3]. However, it is still not clear whether fibrosis directly promotes HCC. Currently, there are very limited therapies for HCC treatment. Therefore, a better understanding of the role of fibrosis and myofibroblast activation in HCC development and progression may provide new therapeutic options for the treatment of HCC.

## 2. The Premalignant and Tumor Microenvironment in HCC

The tumor microenvironment (TME) is defined as the tumor cell population in a complex mixture of surrounding stromal cells, including fibroblasts, endothelial cells, pericytes, immune cells, and proteins like ECM elements, cytokines, chemokines, and enzymes that are secreted by both cancerous and non-cancerous cells [9]. Originally, the TME was not considered to have a role in cancer progression; however, it is now proposed that the stroma is aberrantly activated in cancer and it affects tumorigenesis. In the context of liver cancer, HCC is strongly associated with liver fibrosis and cirrhosis, suggesting that the environment in which HCC rises may influence tumorigenesis. This is different from many other tumors, where fibrosis develops as a reaction of tumor formation [10]. Therefore, it was recently proposed that the premalignant microenvironment (PME) and TME in HCC should be differentiated [10]. PME is characterized by chronic liver injury, inflammation, and fibrosis, and precedes tumor formation, whereas TME evolves in the already developed tumor.

### 2.1. Premalignant Microenvironment in HCC

**Figure 1:**
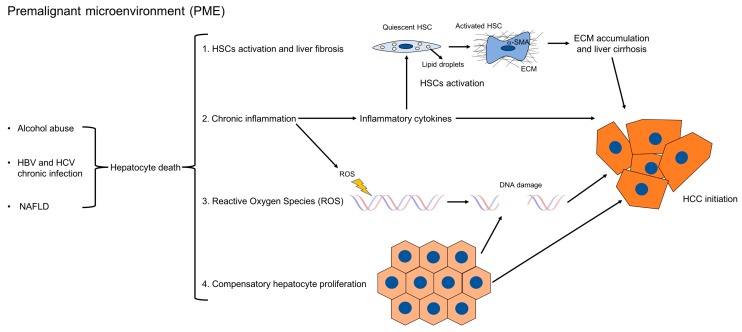
Mechanisms which promote HCC formation in PME. In PME, chronic liver injury causes hepatocyte death, which triggers inflammation, the activation of hepatic stellate cells into ECM-producing myofibroblasts, compensatory hepatocyte proliferation, the release of reactive oxygen species (ROS), and DNA damage. Continuous cycles of this destructive–regenerative process, which precedes tumor formation, are proposed to give rise to replication-related mutations in hepatocytes and eventually HCC. The links between the different mechanisms are indicated here.

Several mechanisms have been proposed to promote tumor formation in PME (Figure 1). First, chronic liver injury causes hepatocyte cell death. It has been demonstrated in mice that abolishing the expression of antiapoptotic proteins such as Nemo, Tak1, Mcl-1, or Bcl-xl, specifically in hepatocytes, increased hepatocyte apoptosis, fibrosis, and consequently, HCC development [11,12,13,14,15]. Accordingly, studies in chronic HBV and HCV patients have shown that elevated levels of ALT, which reflect hepatocyte death, positively correlate with the risk of developing HCC [16,17]. In this setting, hepatocyte death in turn triggers several other mechanisms, such as compensatory hepatocyte proliferation, liver fibrosis, inflammation, the increased generation of reactive oxygen species (ROS), and DNA damage. Hepatocyte proliferation is the consequence of injury-induced necrosis. Continuous cycles of this destructive–regenerative process are proposed to give rise to replication-related mutations in hepatocytes [18] and eventually HCC.

Fibrosis is the main feature of hepatic PME [3]. Liver fibrosis starts as a protective wound healing response to acute liver damage. However, if the injury persists, fibrosis becomes chronic and dysfunctional [19]. Morphologically, liver fibrosis is characterized by the accumulation of ECM, followed by the formation of fibrous scar and subsequent cirrhosis [19,20]. HSCs are the main ECM-producing cells in the injured liver [21]. In healthy livers, quiescent HSCs localize in the space of Disse, function as pericytes, and store vitamin A. However, following continuous liver injury, HSCs activate into myofibroblasts; express alpha-smooth muscle actin (α-SMA); migrate to the site of tissue repair; and secrete ECM, chemokines, and cytokines. In the normal liver, ECM is formed by collagen type IV and VI; however, in fibrotic livers, there is a shift towards the accumulation of fibrillar collagens like type I and III, along with an increased deposition of non-collagenous glycoproteins like fibronectin, undulin, laminin, hyaluronan, elastin, and proteoglycans [20]. Moreover, the deposition of ECM is accompanied by a reduction in the activity of ECM-degrading matrix metalloproteinases (MMPs), favoring the formation of the fibrotic scar [22]. Several human studies have shown that a high fibrosis index and liver stiffness, which are indirect measurements of liver fibrosis, positively correlate with HCC risk [23,24,25]. Moreover, it was also demonstrated that liver fibrosis is linked to increased HCC recurrence after curative resection [26,27,28,29].

Another important feature of hepatic PME is inflammation, which, like fibrosis, is part of the protective wound healing response to acute liver damage. In the short-term, inflammation is believed to be beneficial, eliminating pathogens and favoring liver regeneration. However, chronic inflammation is detrimental and is linked to fibrosis, cirrhosis, and HCC. In fact, HSCs can be activated by several cytokines and growth factors, which are secreted by immune cells, including Kupffer cells, bone marrow-derived monocytes, Th17 cells, and innate lymphoid cells (ILC). Those inflammatory cytokines have been shown to modulate hepatic fibrogenesis in vivo and in vitro [30]. Proinflammatory mediators that have a role in HCC development include IL-1, IL-6, TNF-α, and IL-17 [31,32]. Additionally, secreted cytokines and growth factors can promote proliferative and anti-apoptotic signals in epithelial and tumor cells or induce angiogenesis, therefore favoring tumorigenesis. Interestingly, neutrophils and IL-1 promote hepatocarcinogenesis, but have a limited role in hepatic fibrosis [18,33,34,35]. Although IL-6 is reported to protect against liver fibrosis, it contributes to HCC development [36,37,38,39]. An additional effect of the recruitment of inflammatory cells in the PME is the production of ROS by activated macrophages, activated HSCs, and neutrophils. ROS not only promote fibrosis by facilitating HSCs activation and migration [40], but can also directly induce cancer by generating DNA damage and mutations in hepatocytes [41], or by causing the selective loss of CD4+ T lymphocytes, which mediate tumor immunosurveillance [42]. Consequently, it has been reported that antioxidants which inhibit ROS formation can effectively reduce hepatocarcinogenesis [43,44].

### 2.2. Tumor Microenvironment in HCC

**Figure 2:**
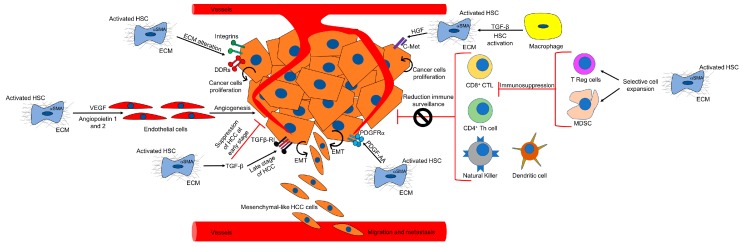
TME in HCC. The figure outlines the different components of the TME and the CAF-dependent mechanisms of hepatocarcinogenesis. (See text for details).

TME in HCC consists of a dynamic network of non-tumoral stromal cells, including cancer-associated fibroblasts (CAFs), B and T cells, neutrophils, endothelial cells, and tumor-associated macrophages (TAMs) (Figure 2). Interestingly, it has recently been shown that, upon liver injury, the expression of adenine dinucleotide phosphate (NADPH) oxidase 1 (NOX1) by macrophages promotes hepatocarcinogenesis by inducing the production of inflammatory cytokines [45]. In addition to these cellular components, the TME is also characterized by profound ECM remodeling [46]. Altogether, the TME interacts bidirectionally with the tumor, generating a tumor-permissive niche. In the following paragraphs of this review, we will provide a detailed overview of CAFs and how they contribute to the development of HCC. TAMs and the other components of the TME are beyond the scope of this review.

## 3. Cancer-Associated Fibroblasts (CAFs)

### 3.1. Origin of CAFs

Fibroblasts were first described by Virchow and later Duvall in 1858 as spindle-shaped cells of the connective tissues that produce collagen. Later in 1971, Giulio Gabbiani showed that fibroblastic cells with contractile properties, called myofibroblasts, may be involved in wound healing [47]. Several studies using genetic cell fate mapping have provided strong evidence that the major precursors of α-SMA-expressing myofibroblasts in most types of experimental liver diseases are HSCs [48,49,50]. Therefore, CAFs most likely derive from HSCs. However, some controversies remain and, other than HSCs, the proposed sources of myofibroblasts are parenchymal cells undergoing epithelial-mesenchymal transition (EMT), bone marrow (BM)-derived cells, mesothelial cells, and portal fibroblasts (PFs). 

Epithelial cells line the surfaces of the body and are located in all organs. EMT is a process in which epithelial cells lose their polarity, acquire a migratory capacity, and become myofibroblasts. Although some studies have shown that hepatocytes and cholangiocytes upregulate α-SMA and suppress epithelial markers under prolonged in vitro culturing [51,52], elegant lineage-tracing experiments have demonstrated that myofibroblasts found in experimental liver fibrosis do not originate from epithelial cells [53,54,55]. These results therefore suggest that myofibroblasts do not originate from EMT in fibrogenesis in vivo [56].

Two BM-derived cells which may potentially become myofibroblasts are mesenchymal stem cells (MSCs) and fibrocytes. MSCs are multipotent cells that can give rise to several cell types, including adipocytes, myocytes, chondrocytes, and osteoblasts. However, recent studies have shown that MSCs may actually have antifibrotic properties and provide a protective microenvironment in the recruited tissue [57].

In contrast, some studies have suggested that in a number of solid tumors, myofibroblasts can originate from the bone marrow [58,59,60,61]. Fibrocytes are cells with a spindle-like shape that were first described in 1994 [62]. They are characterized by co-expressing fibroblast markers (collagen type I, vimentin, and fibronectin) and hematopoietic cell markers (CD45, CD34, MHCII, CD11b, Gr-1, CD54, CD80, CD86, CCR2, CCR1, CCR7, CCR5) [63,64]. Studies have suggested that fibrocytes are recruited to the liver in response to both cholestatic and carbon tetrachloride (CCl_4_)-induced liver injury, where they can differentiate into α-SMA+ myofibroblasts with a contribution range of between 3% and 50% [65,66,67].

Mesothelial cells form a monolayer of specialized cells which line the body’s serum cavities and internal organs. They originate from the embryonic mesoderm layer and have features similar to epithelial cells. Cell fate mapping has demonstrated that during embryonic development, mesothelial cells can give rise to both PFs and HSCs [68,69]. However, it is not clear whether they can be a source of myofibroblasts in liver fibrosis. Interestingly, Asahina and coworkers have shown that mesothelial cells differentiate into both HSCs and myofibroblasts after CCl_4_-induced liver injury, whereas in cholestatic liver injury, they only differentiate into HSCs, not myofibroblasts [70,71]. However, a recent study has suggested that mesothelial cells may have a role in fibrosis of the liver capsule [67].

Portal fibroblasts are a heterogenous population and reside underneath the bile duct epithelium. Since markers which can discriminate fibroblasts from other mesenchymal cells are lacking, it is challenging to identify or purify quiescent PFs. However, activated PFs were first described in cholestatic liver disease by electron microscopy, histology, and immunohistochemistry [72,73,74]. Cell phenotyping has demonstrated that during experimental biliary fibrosis, PFs differentiate into α-SMA-expressing myofibroblasts that produce ECM [75,76,77]. A study proposed that markers such as elastin, Thy1, and Ntpdase2, were specifically expressed by murine PFs, but not by HSCs [78]. The work of Iwaisako et al., using collagen promoter-driven green fluorescent protein (GFP) transgenic mice, has identified two myofibroblast populations: Vitamin A-positive HSCs and Vitamin A-negative PFs [79]. The unifying proposal is that in CCl_4_-induced liver fibrosis, myofibroblasts mainly derive from HSCs, whereas in early cholestatic injury, PFs constitute the major source of myofibroblasts. However, in later cholestatic disease, HSCs again give rise to the majority of myofibroblasts. A novel signaling pathway involving the interaction of mesothelin with a MUC16-Thy1-TGFβRI complex regulates TGF-β1-mediated activation of PFs during cholestatic liver fibrosis [80].

In summary, current studies regarding the origin of hepatic myofibroblasts indicate that, depending on the type of liver injury, they mostly arise from liver-resident HSCs and to a lesser extent, from activated PFs. Mesothelial cells contribute to capsular fibrosis, whereas the contribution to liver fibrosis from BM-derived cells is quantitively small.

### 3.2. Markers of CAFs

In order to study and detect CAFs in the tumor, a specific marker is needed. However, a unique marker for CAFs has not been found. Several markers have been proposed to identify CAFs (Table 1); nonetheless, most of them are not unique to CAFs. For example, α-SMA is widely recognized as a robust CAFs marker [81]; however, it is also expressed by myofibroblasts [82] and its expression may vary between different CAF subtypes [83]. Another CAFs marker is the membrane-bound serine protease fibroblast activation protein α (FAPα), which is upregulated in the majority of epithelial carcinomas [84]. However, it has been shown that FAPα is also not specific to CAFs [85]. Recently, it has been demonstrated that FAPα is expressed in a certain sub-population of CAFs, but absent in others [86]. Fibroblast specific protein 1 (FSP-1) is another CAFs marker [87,88], which is also present in epithelial cells undergoing EMT [89] and in bone marrow-derived cells [90]. Additional proteins expressed in some CAFs include tenascin-C [91], periostin [92], neuron-glial antigen-2 (NG2) [93], podoplanin [94], and the novel identified marker microfibril associated protein 5 (MFAP5) [95]. In summary, the expression of CAFs markers is very heterogenous and it depends on the CAFs subpopulation being analyzed. Therefore, the discovery of CAF-specific markers will be vital to identify and therapeutically target this cell population.

### 3.3. CAFs in HCC

α-SMA-positive myofibroblasts are found in both human and murine HCC. For example, analysis by immunohistochemical technique of liver biopsy specimens from eight patients with HBV-related cirrhosis and HCC demonstrated that desmin-positive and α-SMA-positive cells were present in the perisinusoidal space and between tumor cells [96]. These results were confirmed by another study where liver specimens resected from 24 patients with HCC were analyzed by electron microscopy and immunohistochemistry. Interestingly, stromal cells strongly positive for α-SMA were found between endothelial cells and trabeculae of cancer cells [97]. Moreover, in vivo experiments demonstrated that the majority of cells producing collagens in human HCC were myofibroblasts [98]. In vitro experiments conducted in the same study then showed that HCC cell lines like HepG2, HuH17, and Hep3B, can increase ECM deposition in myofibroblasts by releasing a soluble mediator in the conditioned medium.

Multiple clinical studies have investigated the correlation between the presence of α-SMA-positive myofibroblasts and prognosis after HCC resection. For example, in 130 HCC cases, it was observed that the presence of peritumoral-activated HSCs positively correlates to poor clinical outcome after curative resection [28]. Other studies have confirmed these observations and suggested that metastasis was increased in patients expressing HSC signature genes [26,29,99,100]. Several in vitro and in vivo studies have demonstrated that HSCs can support the growth of HCC cell lines. For example, it was shown that conditioned media from human primary HSCs induced the proliferation and migration of human HCC cell lines cultured in monolayers [101], and similar results were also observed in a three-dimensional spheroid coculture system. In the same study, co-injection of HSCs and HCC cells into nude mice increased tumor growth and invasiveness, and inhibited necrosis. Another study using conditioned media from culture-activated rat HSCs and McA-RH777 rat HCC generated similar results [102]. Further in vitro studies demonstrated that activated CAFs repressed apoptosis in the Huh7 cell line by increasing the Bcl-2/BAX ratio through SDF-1/CXCR4/PI3K/AKT signaling [103]. Another work tried to discriminate the effect of human primary CAFs and primary non-tumoral fibroblasts (NTFs) on human HCC cell lines. The co-culture experiments demonstrated that CAFs up-regulated gene expressions of TGF-β1 and the fibroblast-activated protein (FAP) of HuH-7 and JHH-6, while NTF did not induce the expression of either gene [104]. Interestingly, it was also shown by co-culturing human hepatoma cells and activated human HSCs that the crosstalk between these cells is bi-directional, causing an increased expression of proinflammatory cytokines in hepatoma cells and an increased expression of VEGF and MM9 in HSCs [99]. It was demonstrated that there is a positive feedback loop between CAFs and the forkhead box Q1 (FOXQ1)/N-myc downstream-regulated gene 1 (NDRG1) axis, which drives HCC initiation [105]. Several in vivo studies have confirmed the results observed by co-culture experiments. Subcutaneous co-transplantation of an HSC cell line with MIM-R hepatocytes promoted tumor progression by inducing autocrine TGF-β signaling and nuclear β-catenin accumulation in neoplastic hepatocytes [106]. Similarly, when co-transplanted into nude mice, the HSC cell line LX2 promoted the growth of HepG2 tumors by increasing proliferation and angiogenesis and reducing HepG2 apoptosis [107]. In another study, T6 HSCs orthotopically co-injected into the livers of nude mice, together with H22 HCC cells, increased the tumorigenicity and invasiveness of the cancer cells by promoting angiogenesis [108]. Overall, evidence presented by such studies suggests that CAFs/HSCs are positive regulators of HCC. However, a recent study showed that HSCs may limit HCC progression though the orphan receptor endosialin, which may negatively regulate hepatotropic cytokines, including IGF2, RBP4, DKK1, and CCL5 [109]. This study supports the increasing recognition that HSCs not only have pro-tumorigenic functions, but may also inhibit cancer growth. For example, depleting CAFs in experimental pancreatic ductal adenocarcinoma promoted tumorigenesis [110,111].

## 4. CAF-Dependent Mechanisms of Hepatocarcinogenesis

Several CAF-dependent mechanisms support tumor growth in the liver (Figure 2). For example, CAFs can change the ECM stiffness and in turn affect tumorigenesis. Moreover, CAFs secrete cytokines and other factors which may promote tumor growth, tumor angiogenesis, and epithelial to mesenchymal transition (EMT). CAFs have also been shown to indirectly affect HCC by cross talking with immune cells and reducing immune surveillance.

### 4.1. CAF-Dependent Alteration of ECM Promotes HCC

In the injured liver, activated HSCs secrete ECM proteins and there is a shift towards the accumulation of fibrillar collagens like type I and III. In this altered biomechanical environment, the ECM components can interact directly and indirectly with both cancer cells and stromal cells to change their functions [112]. For example, it was shown that laminin-5, one of the components of the ECM, secreted by primary cultures of human HSCs, stimulated cell migration in several HCC cell lines by activating the MEK/ERK pathway [113]. Moreover, the increase and reorganization of ECM created a stiff microenvironment in the liver. Interestingly, Schrader and collaborators have used “mechanically tunable” matrix-coated polyacrylamide gels to show that an increase in matrix stiffness promoted the proliferation of Huh7 and HepG2 cell lines through the PKB/Akt pathway. In contrast, a soft environment favored cellular dormancy and stem cell characteristics in HCC [114]. Another study using polyacrylamide supports of different stiffnesses suggested that HSCs are also affected by the stiff environment. In fact, primary rat HSCs required a mechanical stiff substrate to differentiate into myofibroblasts [115]. Similar results were also observed when studying CAFs in breast cancer [116]. Of importance, several studies which have measured liver stiffness by using elastography in patients with chronic liver diseases, have confirmed that stiffness correlates with the risk of HCC [24,25,117,118]. The mechanical stress caused by alteration of the ECM is transmitted to the nearby cells by integrins and discodin domain receptors (DDRs), which are responsible for mediating “outside-in” and “inside-out” signaling between ECM and the cells [119]. A study using PDGFC transgenic or Pten null mice as HCC models has shown that several collagen types and integrins were both up-regulated in tumors in these mice, suggesting a correlation in the expression of HCC-associated ECM proteins and ECM-integrins networks [120]. In accordance with these results, integrin β1 and integrin α6 were upregulated in liver biopsies of HCC patients, and the integrin expression positively correlated with pathological grade [121,122]. Integrins have been shown to promote cell proliferation by activating the MAPK and Pi3K pathways, and cell survival through antiapoptotic signaling [123]. Therefore, the altered ECM present in the HCC microenvironment may interact with integrins expressed in hepatocytes, promoting tumor proliferation, migration, and invasion. Like integrins, DDR2 expression was increased in several HCC cell lines and in 112 biopsies from HCC patients, and it was correlated with clinicopathological features of poor prognosis [124]. DDR2 was shown to facilitate HCC invasion and metastasis through activation of the ERK pathway and stabilization of the EMT marker SNAIL1, and this signaling cascade was induced by collagen type I [124]. ECM degradation by MMPs is another key process in the injured liver, which can affect tumorigenesis by releasing growth factors or generating cleavage fragments [125]. Several studies have shown that MMPs can promote tumor cell proliferation, progression, and invasion [126,127,128,129].

### 4.2. CAFs and Tumor Angiogenesis

Tissue hypoxia and vascular disorganization are typically observed in the injured liver. Hypoxia inhibits liver regeneration and promotes angiogenesis, fibrogenesis, and hepatocarcinogenesis [130]. Angiogenesis is the physiological process through which new blood vessels form from pre-existing vessels. Vascular endothelial growth factor (VEGF) is crucial for angiogenesis and it has been shown that it is secreted by both primary and immortalized rat hepatic stellate cells after hypoxic injury [131]. Thus, induction of VEGF may be important in the pathogenesis of liver injury and hepatocarcinogenesis. Another angiogenic factor called angiopoietin-1 was increased in a human fibrotic liver, and was expressed and secreted by activated HSCs isolated from fibrotic mice which were treated by CCl_4_ or underwent bile duct ligation (BDL) surgery [132]. Another study observed that the expression of angiopoietin-1 and angiopoietin-2 was upregulated in HCC patients and correlated with tumor dedifferentiation and tumor vascularity. Moreover, the same study showed that angiopoietin-1 and angiopoietin-2 can be detected in hepatoma cells, HSCs, and smooth muscle cells [133]. Angiopoietein-2 was also found to be upregulated at both mRNA and protein levels in patients with chronic hepatitis B, suggesting that it may contribute to pathological angiogenesis and HCC progression [134]. Several studies using 3D spheroids, co-culture systems of HSCs with endothelial cells, and subcutaneous xenograft models, have shown that HSCs can promote angiogenesis by producing proangiogenic mediators [107,108,135,136].

### 4.3. CAF-Secreted Cytokines

In liver fibrosis, the death of hepatocytes and cholangiocytes causes the activation of HSCs directly or through several cytokines, which are secreted by immune cells. These inflammatory cytokines have been shown to modulate hepatic fibrogenesis in vivo and in vitro [137]. In turn, activated HSCs can produce cytokines that promote cancer proliferation and migration. For example, it was shown that activated HSCs secrete TGF-β, which has a bipartite role. It is a tumor suppressor at early stages of liver damage and regeneration, whereas it acts as a tumor promoter during cancer progression [138], perhaps by inducing nuclear β-catenin accumulation in neoplastic hepatocytes [106]. Studies using transgenic mice have shown that TGF-β-dependent targeting of Snail is required for the formation of liver cancers [139,140]. HSCs also produce hepatocyte growth factor (HGF), which stimulates the motility of Hep3B, HepG2, and Huh7 cells and the migration of primary HCC cells isolated from three patients. HGF promoted phosphorylation of its receptor c-Met and activation of phosphatidylinositol 3-kinase (PI3-K) [141]. A more recent study has shown, by in vitro and in vivo experiments, that HSC-secreted HGF might reduce HCC sensitization to chemotherapeutic agents by promoting epithelial-mesenchymal transition (EMT) and cancer stem cell (CSC)-like properties through the HGF/c-Met pathway [142]. Clinical studies have supported these observations, showing that the expression of HGF and its receptor c-MET was elevated in cirrhotic tissues and in 80% of HCC cases [143].

### 4.4. CAFs and Immune Surveillance

The immune system works as a barrier to tumor formation and progression and several studies have shown that CD8+ cytotoxic T lymphocytes (CTLs), CD4+ Th1 T cells, and natural killer (NK) and dendritic cells (DC) are critical to block tumor development [144,145]. Therefore, both the innate and adaptive immunity contribute to immune surveillance. However, cancer cells can evade the immune system either by producing immunosuppressive factors like TGF-β [146,147] or by recruiting immunosuppressive inflammatory cells such as regulatory T cells (Tregs) or myeloid derived suppressor cells (MDSC), which are able to inhibit the activity of cytotoxic lymphocyte cells [148,149]. It has been shown that populations of Tregs are increased in the tumor and peripheral blood of HCC patients [150]. MDSC in mice express CD11b and Gr-1 and they can be found in the blood, spleen, bone marrow, and tumor microenvironment [149]. In humans, MDSC are characterized by the expression of markers such as CD34, CD33, CD15, and CD16 [151]. Like Tregs, MDSC are also increased in HCC patients [152]. The mechanisms by which Tregs and MDSC limit antitumor immunity have been extensively described previously [153,154] and they are beyond the scope of this review. Using allografts, it was demonstrated for the first time that HSCs can modulate immunity in mice and inhibit T-cell responses by inducing T-cell apoptosis [155,156]. Several studies have shown that CAFs promote HCC by reducing immune surveillance. For example, a cellular transplantation model in immunocompetent mice demonstrated that HSCs prevent T-cell infiltration in tumors, creating an immunosuppressive microenvironment [157]. Immunohistochemical experiments and gene signature analysis in HCC patients have shown that activated HSCs can interact with monocytes, promoting the expression of immunosuppressive cytokines [29]. Moreover, in vivo-activated HSCs caused T-cell hyporesponsiveness, increased T-cell apoptosis, an increased number of immunosuppressive Treg cells, and T-cell mediated cytotoxicity inhibition [158]. Similarly, using the mouse hepatoma cell line H22 together with primary activated HSCs in an orthotopic liver tumor mouse model, it was demonstrated that HSCs increase the number of MDSCs in HCC [159]. More recently, another study confirmed these results and suggested that HSCs induce MDSC by the secretion of prostaglandin E2 (PGE2) [160]. Moreover, it was also suggested, by using co-culture studies, that HSCs promote the conversion of blood monocytes into MDCS in a CD44-dependent manner [161].

### 4.5. CAFs and EMT

EMT is a biological process in which epithelial cells lose their apicobasal polarity, thus allowing them to travel through the ECM like mesenchymal cells [162]. Cells undergoing EMT acquire increased invasiveness, enhanced production of ECM, and more resistance to apoptosis. There are three types of EMT: type 1 gives rise to primary parenchymal cells during embryogenesis; type 2 occurs during wound healing and organ fibrosis; and type 3 modifies the phenotype of cancer cells and is associated with tumor intravasation, migration, and metastasis [163,164]. Several signals, such as TGF-β, epidermal growth factor (EGF), and PDGF, produced by the tumor stroma and in particular by CAFs, may be implicated in EMT [163]. In particular, TGF-β, which signals through both Smad-dependent and -independent pathways, is considered to be the main EMT promoter in epithelial cells, including hepatocytes [165]. Thus, it was shown that TGF-β induces EMT in Ras-transformed hepatocytes [166]. Results from an in vivo HCC model where HCC cells were co-injected with myofibroblasts, and from an in vitro model with a micro-organoid HCC, suggested that the hepatic tumor-stroma crosstalk promotes tumor growth and EMT through a TGF-β and PDGF signaling axis [167].

Other studies have suggested that the TGF-β pathway may be important in the maintenance of self-renewal and pluripotent stem cells, which replicate and generate non-stem differentiated cells. It has been proposed that HCC can originate from a small subset of cancer stem cells, which are transformed from a hypothetical normal stem cell niche [168,169] or from differentiated hepatocytes [170]. Interestingly, experiments performed in rats have suggested that EMT due to chronic TGF-β stimulation produces cancer stem cells from hepatic progenitor-like cells. The same study also showed that pharmaceutical inhibition of microRNA-216a/PTEN/Akt signaling could be a novel strategy for HCC prevention [171]. Another study which employed six different human HCC cell lines has shown that tumor cells with a mesenchymal-like phenotype are refractory to sorafenib-induced cell death [172]. Therefore, these results suggest that EMT induced by TGF-β signaling derived from the tumor stroma may play an important role in supporting tumor growth, and in the generation of chemo-resistant cells, which have stem-like features in HCC.

## 5. Conclusions 

CAFs are one of the most important components of the tumor microenvironment in HCC. Although several studies have shown different mechanisms by which those cells affect HCC growth, this area requires more study. Although the majority of studies presented in this review suggest that CAFs in HCC are positive regulators of cancer, CAFs have been shown to act both as a positive and negative regulator of tumorigenesis in different types of cancer [173]. Therefore, an open question is whether CAFs may somehow have a protective effect and prevent tumor growth in HCC as well. Further studies aiming to answer this question may be important for designing innovative therapeutic approaches. For example, CAFs may be pharmacologically targeted to secrete anti-tumor factors, which might hinder HCC growth and progression. It is also important to understand the origin of CAFs, although HSCs are considered to be the main source. Identification of CAF-specific markers will be crucial to discriminate them from HSCs in the PME and TME of HCC, and to specifically target this cell population.

## Figures and Tables

**Table 1:** Markers of CAFs.

CAFs Markers	References
α-SMA	[81]
FAPα	[84]
FSP-1	[87,88]
Tenascin-C	[91]
Periostin	[92]
NG2	[93]
Podoplanin	[94]
MFAP5	[95]

## Abbreviations

HCC	Hepatocellular carcinoma
HSCs	Hepatic stellate cells
CAFs	Cancer-associated fibroblasts
PFs	Portal fibroblasts
ECM	Extracellular matrix
EMT	Epithelial-mesenchymal transition
PME	Premalignant microenvironment
TME	Tumor microenvironment
HBV	Hepatitis B virus
HCV	Hepatitis C virus
NAFLD	Non-alcoholic fatty liver disease
α-SMA	Alpha-smooth muscle actin
MMPs	Matrix metalloproteinases
ROS	Reactive oxygen species
TAMs	Tumor-associated macrophages
NADPH	Nicotinamide adenine dinucleotide phosphate
NOX1	NADPH oxidase 1
CCl_4_	Carbon tetrachloride
BDL	Bile duct ligation
FAP	Fibroblast activated protein
NTF	Non-tumoral fibroblast
VEGF	Vascular endothelial growth factor
EGF	Epidermal growth factor
DDRs	Discodin domain receptor
PI3-K	phosphatidylinositol 3-kinase
HGF	Hepatocyte growth factor
CSC	Cancer-stem cell
FAPα	Fibroblast activation protein α
FSP-1	Fibroblast specific protein 1
NG2	Neuron-glial antigen-2
MFAP5	Microfibril associated protein 5
Treg	Regulatory T cell
MDSC	Myeloid derived suppressor cell
DC	Dendritic cell
NK	Natural killer
CTLs	Cytotoxic T lymphocytes
MSCs	Mesenchymal stem cells
GFP	Green fluorescent protein
